# Neural Crest Does Not Contribute to the Neck and Shoulder in the Axolotl (*Ambystoma mexicanum*)

**DOI:** 10.1371/journal.pone.0052244

**Published:** 2012-12-27

**Authors:** Hans-Henning Epperlein, Shahryar Khattak, Dunja Knapp, Elly M. Tanaka, Yegor B. Malashichev

**Affiliations:** 1 Department of Anatomy, University of Technology Dresden, Dresden, Germany; 2 Center for Regenerative Therapies, University of Technology Dresden, Dresden, Germany; 3 Department of Embryology, Faculty of Biology and Soil Sciences, Saint-Petersburg State University, St. Petersburg, Russia; 4 Department of Vertebrate Zoology, Faculty of Biology and Soil Sciences, Saint-Petersburg State University, St. Petersburg, Russia; University of Otago, New Zealand

## Abstract

**Background:**

A major step during the evolution of tetrapods was their transition from water to land. This process involved the reduction or complete loss of the dermal bones that made up connections to the skull and a concomitant enlargement of the endochondral shoulder girdle. In the mouse the latter is derived from three separate embryonic sources: lateral plate mesoderm, somites, and neural crest. The neural crest was suggested to sustain the muscle attachments. How this complex composition of the endochondral shoulder girdle arose during evolution and whether it is shared by all tetrapods is unknown. Salamanders that lack dermal bone within their shoulder girdle were of special interest for a possible contribution of the neural crest to the endochondral elements and muscle attachment sites, and we therefore studied them in this context.

**Results:**

We grafted neural crest from GFP+ fluorescent transgenic axolotl (*Ambystoma mexicanum*) donor embryos into white (*d/d*) axolotl hosts and followed the presence of neural crest cells within the cartilage of the shoulder girdle and the connective tissue of muscle attachment sites of the neck-shoulder region. Strikingly, neural crest cells did not contribute to any part of the endochondral shoulder girdle or to the connective tissue at muscle attachment sites in axolotl.

**Conclusions:**

Our results in axolotl suggest that neural crest does not serve a general function in vertebrate shoulder muscle attachment sites as predicted by the “muscle scaffold theory,” and that it is not necessary to maintain connectivity of the endochondral shoulder girdle to the skull. Our data support the possibility that the contribution of the neural crest to the endochondral shoulder girdle, which is observed in the mouse, arose *de novo* in mammals as a developmental basis for their skeletal synapomorphies. This further supports the hypothesis of an increased neural crest diversification during vertebrate evolution.

## Introduction

The vertebrate shoulder girdle is a complex structure consisting of endochondral and dermal skeletal elements. The endochondral part of the shoulder girdle and the limb skeleton were long considered to be derived solely from lateral plate mesoderm, whereas the dermal part of the shoulder girdle was regarded as neural crest derived. These assumptions were commonly accepted, but barely experimentally verified. Experiments with quail-chick chimeras were the first that established a somitic contribution to the endochondral scapula in addition to the material of the lateral plate mesoderm, which forms the rest of the endochondral shoulder girdle [1], [2]. The embryonic origin of the dermal shoulder elements was not precisely determined, although often inferred a priori as neural crest derived. For example, the mesodermal origin of the dermal clavicles in birds suggested on the basis of quail–to–chick mesoderm transplantations [1], was in an apparent contradiction to these expectations [3]. Nevertheless, the actual contributions of mesoderm and neural crest to this bone appear different [4]. In particular, the caudally migrating neural crest cells from the level of rhombomeres 6 and 7 and somites 1 and 2, form the cranialmost medial part of the dermal clavicle, e.g., connective tissue at the site of attachment of the cleidohyoid muscle, which connects the clavicle to the tongue skeleton [4], [5]. However, the embryonic origin of the rest of the avian clavicle has never been thoroughly investigated and the contribution of the neural crest to this or the other bones of the shoulder girdle is not known in most vertebrates.

The relative contribution of neural crest to the endochondral and dermal skeleton in vertebrates varies along the cranio–caudal body axis [3], [4], [6], [7], [8]. In the head, both the endochondral and dermal skeleton arise from neural crest while in the trunk only the dermal bones are of neural crest origin. Deviations from that rule were found in the mouse [9], where the endochondral bones of the shoulder girdle receive contributions from the postotic neural crest: the spine, the acromion, the coracoid process of the scapula, the endochondral part of the clavicle, the manubrium sterni, and connective tissue at corresponding attachment sites for muscles. Such a neural crest contribution to the mammalian shoulder girdle within an otherwise endochondral environment was proposed as an evolutionary remnant of the dermal skeleton as seen in fish and early tetrapods [9]. Matsuoka et al. [9] argued that this neural crest-derived muscle attachment pattern is a trait shared by all gnathostomes with paired fins (formulated as the “muscle scaffold theory”). This theory suggests that the homology of corresponding muscles may serve as an indicator of homology of skeletal elements receiving attachment sites of those muscles. Particularly, based on the muscle connectivity pattern, the dermal cleithrum of fishes and amphibians was homologized to the neural crest population discovered within the endochondral scapula of the mouse [9]. This generalization, and particularly, the idea that the scapula spine of mammals is homologous to the cleithrum (“cell population ghost” of the cleithrum) of amphibians has been widely debated [10], [11]. Whether the “muscle scaffold theory” and its consequences are indeed correct or not, depends on whether the cranial aspect of the endochondral, and not only dermal shoulder girdle of other taxa also includes contributions from the neural crest. Studying the origin of the shoulder girdle in salamanders may be critical for addressing this issue, because their shoulder girdle lacks any traces of dermal bones [12], [13].

**Figure 1:**
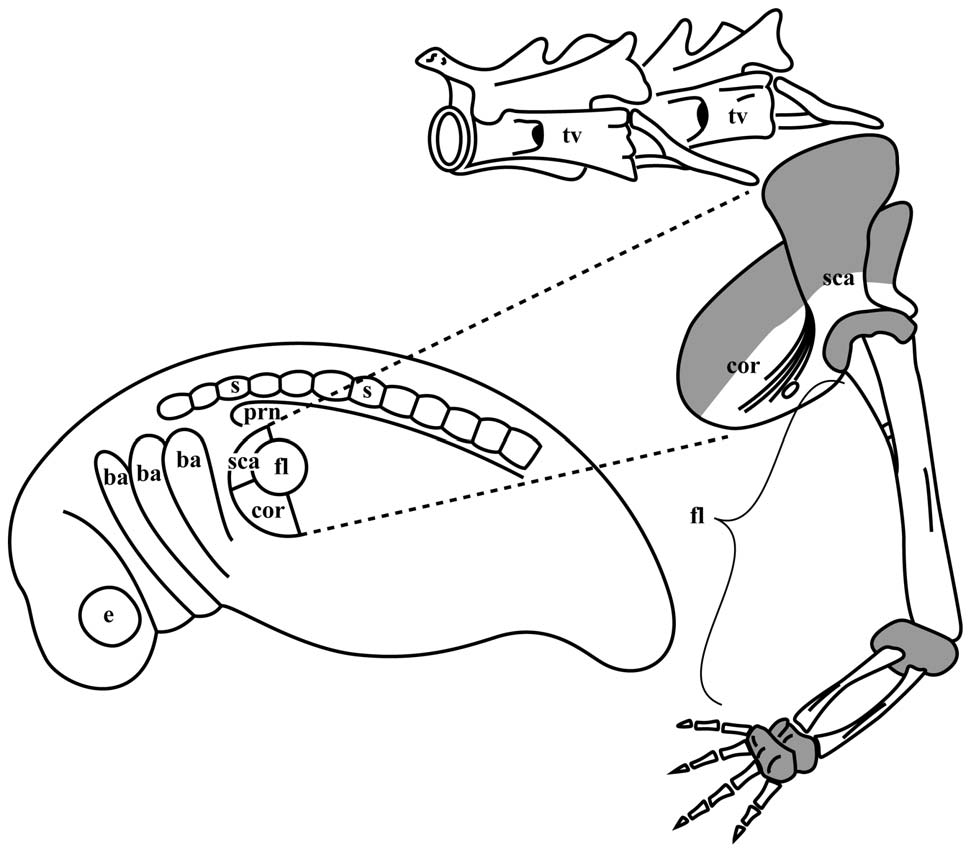
Relations of the shoulder girdle to the embryonic and adult anatomy. According to the fate map by Stocum and Fallon [32], the shoulder girdle of the axolotl arises mainly from flank mesoderm as part of the embryonic limb field (left). The upper, scapular (sca), and the lower, coracoid (cor) parts of the shoulder girdle (right) originate from the specific areas of the limb field around the region, which gives rise to cartilage and connective tissues of the prospective free limb (fl) [32]. The shoulder girdle region is thus positioned just caudal to the branchial arches (ba), where the main streems of migrating neural crest cells pass. In adults, the coracoid plate of one side meets the contralateral counterpart along the ventral midline of the animal, while the upper scapular edge reaches the level of transverse processes of the thoracic vertebrae. These parts of the shoulder girdle are cartilaginous (grey) in the axolotl throughout life, while the middle of the shoulder girdle (both in the scapula and the coracoid plate), from where the limb emerges, are ossified in adults. The anterior, cranial edge of the scapula bears the attachment sites of muscles (m. cuccularis, m. opercularis), which connect the shoulder girdle to the occipital bones of the skull. Other abbreviations: e, eye; prn, pronephros; s, somite, tv, thoracic vertebrae. Not to scale.

Here we examined the contribution of neural crest to the shoulder girdle in the axolotl (*Ambystoma mexicanum*). Anatomically, the shoulder girdle arises as a part of the limb field mesoderm of the flank just behind the branchial arches, where the main streems of migrating neural crest cells pass to form hyobranchial cartilages (Fig. 1). The availability of GFP+ transgenic axolotls [14] allowed a set of transplantation experiments, with which the hypothesis on neural crest contribution to the shoulder girdle in this species could be tested rigorously. Long–term fate mapping was achieved by grafting the neural folds (including the neural crest) from neurulating embryos of GFP–expressing germ–line transgenic axolotls [14] into white (*d/d*) hosts (see “Materials and Methods”). We show that in the axolotl the neural crest does not contribute cells to the muscular–skeletal system of the neck and shoulder girdle including the muscle attachment sites, and conclude that this characteristic contradicts the “muscle scaffold theory”. We therefore propose that the population of neural crest cells that participate in building muscle–skeletal connections of the skull and the endochondral shoulder girdle of the mouse [9] may be a synapomorphy of mammals, which appears long after the earlier population of neural crest cells, that build the dermal shoulder girdle. In axolotl the former is not yet present, while the latter has already disappeared along with the reduction of the dermal bones in salamanders.

## Results

**Figure 2:**
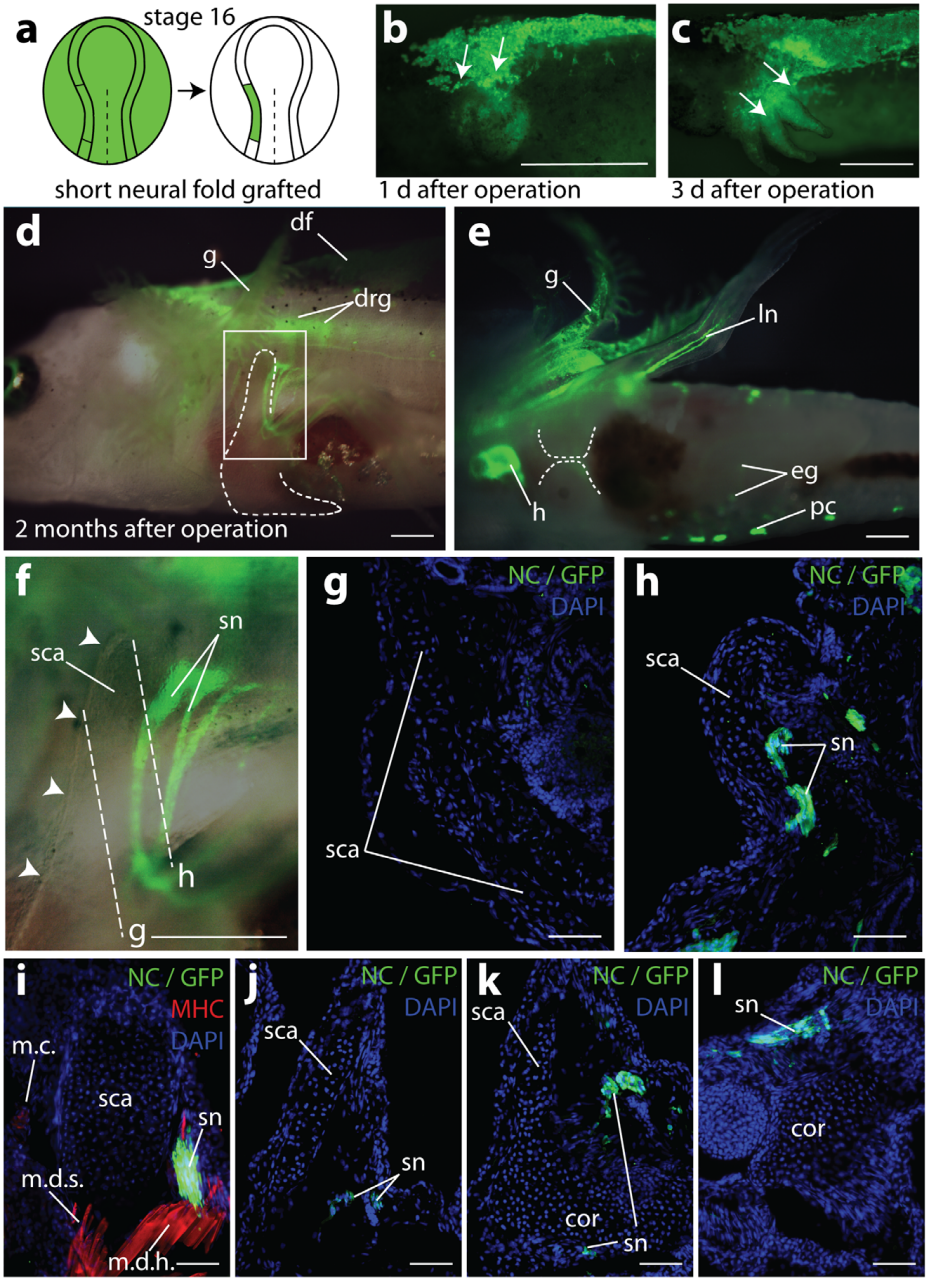
Results of grafting one short left neural fold fragments. **a,** Schematics demonstrating orthotopical grafting of a short left GFP+ neural fold fragment (including neural crest) into a white (d/d) host. The graft is extirpated from a GFP+ neurula (green, stage 16) and extends from a prospective posterior head to an anterior trunk region. It is implanted into a white host where a similarly sized fragment was extirpated previously. **b** and **c,** left flank of white hosts 1 day (**b**) and 3 days (**c**) after the operation. In vivo visualization of GFP+ neural crest cells at an anterior trunk level where they migrate laterally from the top of the neural tube; arrows show the main direction of migration. **d–h**, two months old juvenile carrying a short GFP+ neural fold fragment. No neural crest cells were present in the scapula, or elsewhere in the shoulder girdle. However, all other neural crest derivatives located at this level were GFP+. **d**, left side of operated juvenile where cranial and ventral margins of the GFP negative shoulder girdle are visible through the transparent skin. Girdle cartilage is outlined with a dashed line. **e,** ventral aspect of the juvenile. Gills, nerve fibres in the limb, pigment cells, heart and enteric ganglia are clearly GFP+, while the ventral halves of the cartilaginous coracoid plates (indicated with the dashed line) are GFP negative. **f**, enlarged area of the scapula framed in (**d**). Only spinal nerves of the brachial plexus appear GFP+. The cranial margin of the scapula is marked with white arrowheads. No GFP+ cells are detectable along its cranial margin, where muscles exist that attach it to the skull. **g, h**, transverse sections through the juvenile (sectioning planes see (**f**)) with GFP+ spinal nerves but GFP negative scapular cartilage and connective tissue. **i–l**, sagittal sections through the shoulder girdle region in a 1.5 month old juvenile from dorso-medial (**i**, scapula tip as in **h**) to ventro-lateral (**l**, glenoid region). Anti-Myosin heavy chain-rhodamine immunostaining only in **i**, for better visualization of GFP+ cells. Note GFP+ staining in all sections only in spinal nerves, but not in cartilage or muscle attachment sites of the shoulder girdle. Abbreviations: cor, coracoid; df, dorsal fin; drg, dorsal root ganglia; eg, enteric ganglia; g, gills; h, heart; ln, limb nerves; m.c., musculus cuccularis; m.d.s., musculus dorsalis scapulae; m.d.h., musculus dorsalis humeralis (latissimus dorsi); pc, pigment cells; sca, scapula; sn, spinal nerves. Scale bars: **b**–**f:** 1 mm; **g–l**: 100 µm.

To determine whether neural crest cells contribute to the shoulder girdle in the axolotl, we first grafted the left neural fold (posterior cranial to anterior trunk region) including neural crest cells from a GFP+ donor to a white (d/d) host (Fig. 2a). Our neural fold grafts comprehensively labeled the neural crest, since we observed GFP+ cells in all neural crest derivatives (dorsal fin mesenchyme, melanophores, jaws and pharyngeal arches, dorsal root ganglia, Schwann cells, the truncus arteriosus and septa of the heart, and neurons and glial cells of the enteric nervous system) from mid-head to mid-trunk levels (Fig. 2 b–e). Strikingly, no GFP+ cells were found in the shoulder girdle, neither in the cartilage, perichondrium, or muscle attachment sites (Fig. 2 g–l) including the cranial edge of the scapular blade (Fig. 2 e, f).

**Figure 3:**
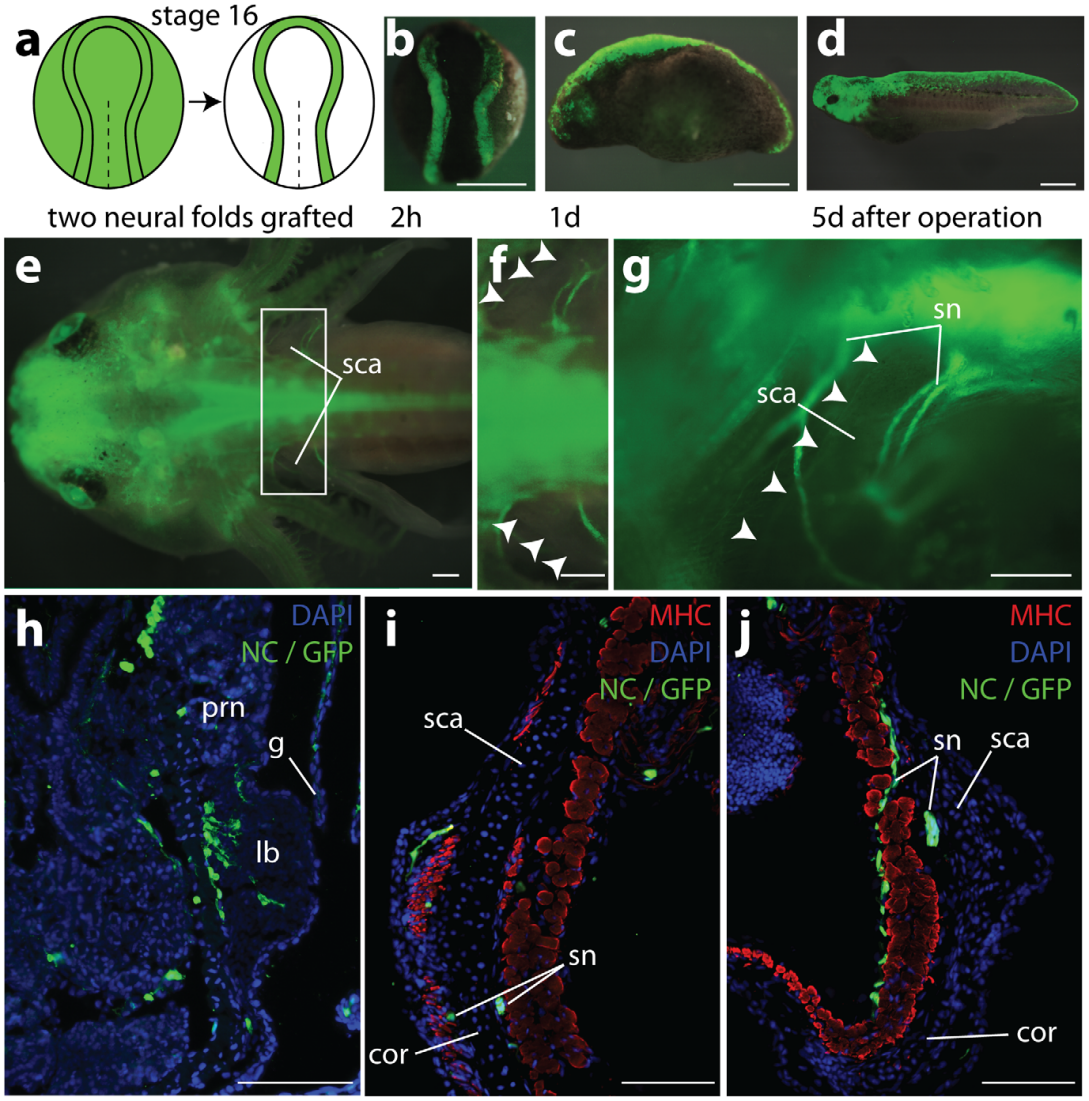
Results of double-sided neural fold transplantations. **a,** Schematics demonstrating grafting of both GFP+ neural folds (including neural crest) from a GFP+ neurula (green, stage 16) into a white (d/d) host. Both entire GFP+ neural folds were grafted into a white host in which the neural folds from both sides had been removed before. **b–d,** embryos containing 2 GFP+ neural folds 2 h, 1 day, and 5 days after the operation, respectively. **e–g**, 2 months old juvenile; all neural crest derivatives are GFP+. **e**, dorsal aspect of the juvenile; scapulae visible on both sides through the skin. **f**, enlargement of area framed in (**e**), the cranial margins of the dorsal scapulae are marked with arrowheads. **g**, the same larva viewed from the left side (head to the left). The scapula blade, visible through the skin between the spinal nerves of the brachial plexus, contains no GFP+ signal, neither within the cartilage nor along the cranial margin (arrowheads). **h**, transverse section through a three weeks old juvenile at the fore-limb bud level. Neural crest cells migrating in a kind of stream-like order are detected at the base of the forelimb bud where they might form sheaths of nerve fibres. **i**–**j**, transverse sections through the middle part of the scapulo-coracoid at two cranio-caudal levels on the left (**i**) and the right (**j**) sides of another 1.5 months old juvenile after double sided neural fold transplantation. Note GFP+ staining in all sections only in spinal nerves, but not in cartilage or connective tissue of the shoulder girdle. Abbreviations: lb, limb bud; other abbr. as in Fig. **1** and **2**. Scale bars: **b–g:** 1 mm, **h–j:** 100 µm.

Since neural crest cells can potentially migrate long distances along the anterior-posterior axis (half the length of the embryo; unpublished observation) and from one left or right fold to the other (up to 30% [15]) we grafted both left and right neural folds including the entire cranial and trunk regions (Fig. 3 a, b). This resulted in labelling of more than 95% of all neural crest cells [16]. Such a strong labeling is visible in Fig. 3 c–e. Thus, with this type of operation the possibility was excluded that unlabelled neural crest cells migrate from distant or closer ipsi- and contralateral sites and contribute to the shoulder girdle randomly. Even when excluding the participation of unlabelled neural crest we could not find GFP+ neural crest cells in the muscle attachment sites or any part of the shoulder girdle cartilage of 1.5–2 month-old juveniles (Fig. 3 e–g, i, j). In younger animals (2–3 weeks) GFP+ neural crest cells were seen as chains of migrating cells at the base of the fore-limb bud (Fig. 3 h), which later occurred here only within the roots of spinal nerves.

**Figure 4:**
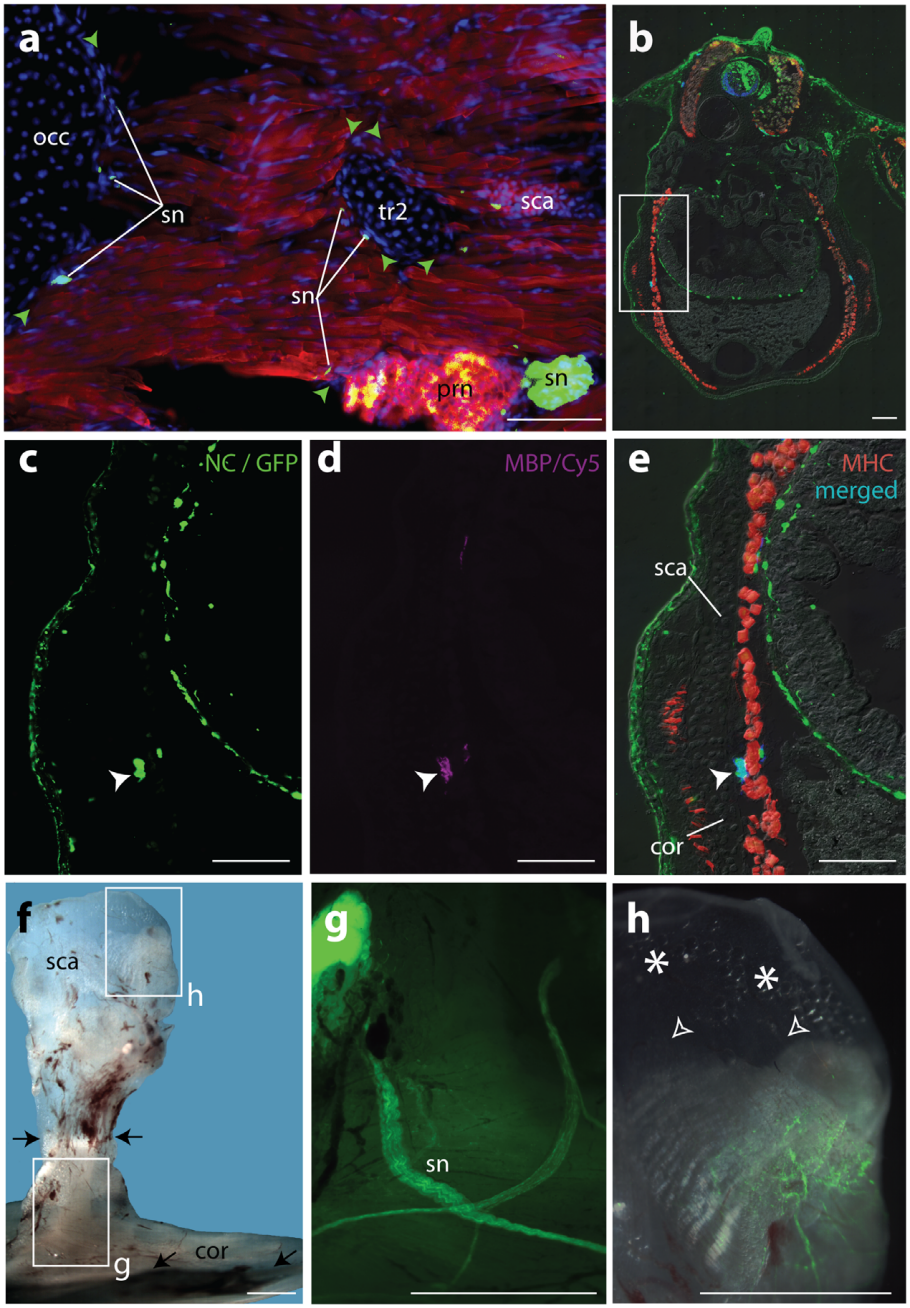
Results of additional experiments. **a**, Sagittal section through the neck epaxial muscles in between the scapular tip and occipital region of the skull; this region is devoid of any neural crest-derived connective tissue. Only intersegmental nerves are present along the intermuscular septae are GFP+ (green arrowheads). **b–e**, transverse sections through the shoulder girdle region of a juvenile (1 month) containing two GFP+ neural folds (see Fig. **3a**). The framed area in (**b**) is enlarged in (**c–e**). **c–e**, GFP+ spinal nerves close to the shoulder girdle cartilage (**c**) and Myelin Basic Protein+ cells (anti-MBP-Cy5 immunostaining) in (**d**) are co-localized (**e**) as indicated with white arrowheads. **f–h,** medial aspect of the right shoulder girdle (soft tissues included) of a mature axolotl (3 years) containing two short GFP+ neural fold fragments on either side (same experiment as in (Fig. **2a**), but with short double- sided graft). **f**, bright field micrograph of an isolated shoulder girdle whole mount with framed areas enlarged in (**g**) and (**h**). The dorsal border of the ossified part of the scapulo-coracoid is indicated with black arrows. **g**, GFP+ spinal nerves over the GPP-negative ossified scapulo-coracoid. **h**, nerve net in the muscles connecting to the scapula. GFP+ cells are not present in muscle attachment sites (empty white arrowheads) and the tip of the scapula of somitic origin (white asterisks). Abbreviations: tr2, transverse process of the second vertebra; occ, occipital bone; other abbr. as in Figs. **1**–**3**. Scale bars: **a–e :** 100 µm, **f–h :** 5 mm.

In both types of experiments we also did not find neural crest cells in the otic capsules or occipital bones of the skull (Fig. 4a), which are of mesodermal origin [7], [17]. Furthermore, all GFP+ cells close to the shoulder girdle were well co-localized with immunostaining for a glial marker, myelin basic protein (Fig. 4 b–e). Since we found no difference between the two types of experiments in the labelling of the neural crest derivatives in the neck and shoulder girdle region of the trunk, we further used double sided, but shorter neural fold fragment transplantations. To examine the possibility that neural crest cells migrate into the shoulder girdle at later stages of development, we also examined 2–3 year old adults (ossified bone) that had received double sided GFP+ neural fold transplants. We found that GFP+ signals were still restricted to nerve fibers of the brachial plexus and to neuronal nets in the muscles, but were not present in the shoulder girdle itself or in muscle attachment sites (Fig. 4 f–h). Altogether, these results show that neural crest does not contribute to the endochondral shoulder girdle in the axolotl.

## Discussion

The complete absence of neural crest cells in the endochondral shoulder girdle of the axolotl contrasts with the apparent contributions of the neural crest to the endochondral shoulder girdle and muscle attachment sites in the mouse [9], [18], where neural crest cells were interpreted to be descendants of the neural crest-derived dermal portion of the ancestral shoulder girdle [9]. However, we have found that the shoulder girdle in axolotl did not retain neural crest derivatives, neither as neural crest-derived cell populations within the endochondral elements nor as connective tissue at muscle attachment sites. This may reflect a more common vertebrate trait where muscle attachment points to the endochondral shoulder girdle have no neural crest, but only a mesodermal contribution. It further suggests that neural crest contribution to endochondral elements of the shoulder girdle and the skull and corresponding attaching muscles is not necessary for normal connections between those parts of the skeleton.

Observations in several other species have further implied that the neural crest does not make a significant contribution to muscle attachments of the endochondral shoulder girdle in those species either. For example, in quail/chick chimeras, neural crest–derived cells were found in the dermal clavicle, but very few to none have been observed in the region near the endochondral scapula [4], [19] (I McGonnell and R Huang, pers. comm., own unpublished observations). In turtles, neural crest marker gene expression (HNK-1, PDGFRα) has suggested that neural crest contributes to the dermal plastron (epiplastrons, homologues of the clavicles of other reptiles, and the entoplastron, a homologue of the interclavicle) and dermal parts of the carapace [7], [8]. The neural crest apparently contributes also to the dermal gastralia in crocodiles [7]. Marker expression was not observed in the endochondral shoulder girdle of crocodiles, which is lacking dermal clavicles. Finally, genetic labelling of zebrafish neural crest using a photoconvertible kikumeGR driven by the *Sox10* promoter so far also did not reveal neural crest derivatives in the endochondral shoulder girdle of this fish species while this technique yields clear labelling of the hyoid and pharyngeal arches (G. Crump, pers. comm.). Taken together, these observations suggest that the axolotl may be not an extreme case but rather that the mouse may be the exception with respect to neural crest participation in the shoulder girdle.

Hence, the transformational scenario suggested by Matsuoka et al. [9] requires reconsideration. As a plausible alternative, we propose that the neural crest population of cells in the endochondral shoulder girdle of the mouse is non-homologous to the cell population that builds the dermal skeleton (e.g., the cleithrum) of ancestral gnathostomes in the neck–shoulder region. During tetrapod evolution, there was a substantial diminution of the dermal skeleton at the head to trunk transition region [4], [20]. In our view, the axolotl illustrates this evolutionary loss of dermal shoulder girdle elements in tetrapods during their emergence from water to land. We therefore suggest that the neural crest population found in mouse rather reflects a secondary broadening of the neural crest diversity that occurred in mammals. New shoulder elements such as the endochondral clavicle, a part of the scapular spine, and the sternal manubrium appear, which represent apomorphic characteristics of the Theria [3], [11], and which mosaically evolved in primitive mammals [21], [22]. These anatomical mammalian innovations could receive new contribution from neural crest rather than co-opting cells from the former dermal skeleton. This idea supports the view that the neural crest proper is an evolving entity and that the number of derived cell types may change during the evolution of vertebrates, with some cell types appearing de novo and some disappearing in particular lineages [15], [23]. For example, the population of neural crest cells, which gave rise to the cleithrum and other dermal bones of the primitive shoulder girdle, has disappeared completely in the axolotl, i.e., neural crest cells were neither found as separate dermal bones nor as cartilage or connective tissue derivatives at the muscle attachment sites. At the same time an evolutionarily younger population of neural crest cells, which later gave rise to mammal–specific shoulder girdle elements, could also not be found yet in the salamander shoulder girdle. Finally, we hypothesize also, that in other phylogenetic groups, which have lost cleithral bones (turtles, birds, and other diapsids) neural crest contribution is unlikely to be found in the endochondral shoulder girdle and particularly in the scapular region, since the “cell population ghost” of the cleithrum does not exist.

## Materials and Methods

### Animals

Adults of the Mexican axolotl (*A. mexicanum*) were bred in the facility of the Max-Planck-Institute of Molecular Cell Biology and Genetics in Dresden [14], [24]. Eggs were kept in tap water at room temperature or, to delay development and synchronize clutches, at 7–8°C. Embryos and larvae were staged according to corresponding normal tables [25], [26].

### Ethics Statement

This study does not include any study of human subjects or non-human primates, thus does not need any specific adherence to the Declaration of Helsinki or Weatherall report. As for the work with other subjects, this work only involved grafting experiments done in early embryos, collection of tissues for fixation, and histological and anatomical analysis; hence this work was done using widely approved methods for treating axolotls to reduce suffering and thus does not require any formal approval by an ethics committee. The European Directive 86/609/EEC states that fetal animals in the third trimester of development are protected by law. This directive, however, does not apply to our study, because the embryos we used were early neurula embryos, i.e., had far from reached the protected development stages.

### Transgenesis and Transgenics

The generation of transgenic animals ubiquitously expressing GFP under the control of the CAGGS promotor has been described previously [14]. This preliminary work included examination at a high resolution the contribution of GFP protein into cells in the forelimb tissues, heart, liver, lungs, and eyes, as well as dorsal fin and tails, limb regenerative blastemas and regenerated tails. All the tissue types ubiquitously expressed GFP+. The only cell type which we found not GFP positive was erythrocytes, showing no detectable GFP protein level at Western blots, probably because of general transcriptional inhibition [27]. Otherwise, the ubiquitous GFP expression was further confirmed by us in an earlier report (see Supplementary Figure 2 and Supplementary Table 1 in [24], http://www.nature.com/nature/journal/v460/n7251/extref/nature08152-s1.pdf), where we carefully documented that all cells are green in the transgenic GFP line, particularly at the forelimb level, both in normal and regenerated tissues.

The GFP transgenic embryos, used as donors for operations, as well as the host embryos, had d/d (white mutant) background. The d/d mutant axolotls were chosen for better visualization of GFP in the cells. Although in the white mutant melanophore migration on the dorsolateral route (between somites and epidermis) is inhibited, the defect is not due to a deficiency in melanophores themselves, but due to a retarded maturation and inability of dorsolateral subepidermal extracellular matrix to support neural crest cell migration. A misexpression of proteoglycan isoforms in the extracellular matrix coincides with the early migration of melanophores and substantially alters the latter [28], [29]. To our knowledge, the dorsolateral pathway in the trunk is used in dark axolotl embryos only by pigment cells (melanophores and xanthophores). The sole migration of melanocytes, but not of other cell types of neural crest origin, on the lateral route of the trunk has long been known also for the chick [30]. Therefore, we confirm here that defects of migration in the white mutant only refer to the lateral migration of pigment cells. Other neural crest derivatives are not affected, justifying the use of *d/d* mutant axolotls for our study.

### Operations on Embryos

Embryos were dejellied in sterile 1× Steinberg solution [31] containing antibiotics (Antibiotic-Antimycotic; Invitrogen, Karlsruhe, Germany). The embryos were then transferred into agar dishes (2% agar in tap water) filled with sterile Steinberg solution and held steady in pits of the agar layer. Operations were carried out with tungsten or preparation needles either in 4× Steinberg solution in order to obtain an optimal separation of tissue layers (epidermis, mesoderm, endoderm) in most cases or in 1× Steinberg solution, when an operation (e.g., grafting long bilateral neural folds) lasted 20–60 min. With hypertonic Steinberg solution tissue layers can be separated more easily, but a longer stay could cause malformations or death of embryos.

### Neural Fold (Neural Crest) Grafting

A unilateral (left) fragment neural fold (n = 10) from the prospective posterior head to anterior trunk neural fold region containing neural crest, or the entire left and right cranial and trunk neural fold of a GFP+ donor (n = 5) were grafted into a white (d/d) host at stage 16 [25] where similar sized neural fold areas had been removed. The implanted fold fragments were pressed against the body of the host with a piece of glass to assist healing. Larvae carrying two grafted folds were carefully examined on ca. 300 transverse and sagittal cryostat sections (see below). There was no evidence of GFP silencing in neural crest derivatives, such as dorsal root ganglia. In addition we investigated both left and right halves of the shoulder girdle of two about 2.5 year old adult axolotls (when the scapulocoracoid is ossified) that had received short left and right neural fold fragments at the neurula stage as described [24].

### Sectioning and Immunostaining

Transverse cryosections (20–25 µm) were cut through the shoulder region of the anterior trunk in about 1.5–2.5 month old juveniles that contained GFP+ tissues. Specimens were fixed with 4% paraformaldehyde at 4°C over night, washed in PBS, incubated in 30% sucrose overnight, infiltrated with 5% gelatine (Merck) overnight, embedded into 7.5% gelatine and frozen on dry ice. Cryosections were stained with primary antibodies against GFP (Invitrogen) to increase the visibility of transgenic donor cells. Alexa 488- conjugated secondary antibodies were used for detecting GFP. Additionally, we used rhodamine-conjugated anti-Myosin heavy chain antibodies (clone 4A.1025, a kind gift from Simon Hughes, Kings College, London) to visualize skeletal muscles and anti-Myelin-basic-protein antibodies (GeneTex) with a secondary antibody conjugated with Cy5. All sections were stained with DAPI, embedded into glycerol-PBS (1∶1) and analysed with epifluorescence microscopes.
